# Indirect X-ray detectors with single-photon sensitivity

**DOI:** 10.1107/S1600577522009584

**Published:** 2022-10-25

**Authors:** Kristof Pauwels, Paul-Antoine Douissard

**Affiliations:** a ESRF – The European Synchrotron, 71 Avenue des Martyrs, Grenoble, France; Paul Scherrer Institut, Switzerland

**Keywords:** X-ray detectors, indirect detection, single photons, scintillation, sCMOS

## Abstract

The new generation of synchrotron light sources are pushing X-ray detectors to their limits. Very demanding conditions with unprecedented flux and higher operating energies now require high-performance X-ray detectors combining sensitivity, efficiency and scalability. Over the years, hybrid pixel detectors have supplemented indirect detectors based on scintillation, with undeniable advantages. Such detectors based on silicon are, however, rather expensive to produce and are no more satisfying in terms of X-ray stopping power when targeting energies above 20 keV. An indirect detector with single X-ray photon sensitivity therefore offers promising opportunities for applications operating over a wide range of energies and fluxes. In this work, the performances of such an approach are investigated with state-of-the-art elements: a commercial sCMOS camera with fiber-optics plate coupling and a Gd_2_O_2_S:Tb powder-based scintillator. A simple method is presented for evaluation of the single X-ray photon detection limit and single X-ray sensitivity is demonstrated with the studied detector above 20 keV. *Geant4* simulations also provide insight to better define the limiting factors. Finally, guidelines are provided for future R&D in the design and assembly of an innovative detector combining advantages of direct and indirect detection schemes.

## Abstract 2

A systematic method for evaluation of the single X-ray photon detection limit is presented and single X-ray sensitivity with a commercial detector is demonstrated above 20 keV.

## 1. Introduction

The extraordinary performances of today’s synchrotron light sources benefit a variety of applications, including material science, medical and pharmaceutical research, biology, and environmental science. They also set drastic constraints in the ability to detect X-rays in more and more demanding conditions. In response, high-performance X-ray detectors are being developed to combine sensitivity, efficiency and scalability as many of the imaging and scattering experiments require an excellent signal-to-noise ratio and a large field of view (>10 cm × 10 cm). Over the years, hybrid pixel detectors (Hatsui & Graafsma, 2015 ▸; Ballabriga *et al.*, 2016 ▸; Förster *et al.*, 2019 ▸) have supplemented the historical flat-panel technology with undeniable advantages. A strong evolution of the detector technology is likely to continue with the fourth generation of synchrotron light sources based on the hybrid-multibend-achromat lattice technology (Raimondi *et al.*, 2021 ▸). The European Synchrotron Radiation Facility (ESRF) recently upgraded its storage ring to become the first of this new generation of synchrotrons in operation: the Extremely Brilliant Source (ESRF-EBS) (Raimondi, 2016 ▸). This upgrade leads to substantial improvements in both coherence and brilliance of the source. As a result, the synchrotron beamlines are able to run experiments at higher X-ray energies and with 10- to 100-fold gain in X-ray flux. This now sets very challenging conditions for the operation of detectors, especially for technologies based on the direct conversion of X-rays into electrons. Above 20 keV, silicon-based technologies are no more satisfying in terms of X-ray absorption efficiency. Hybrid pixel detectors based on high-*Z* materials, such as GaAs and CdTe, can be considered instead. However, these detectors suffer from a non-uniform sensor response (affecting flat-field images), as well as instabilities due to polarization effects under intense X-ray fluxes (Ruat & Ponchut, 2014 ▸; Tsigaridas & Ponchut, 2019 ▸).

The opportunity to consider again indirect detection schemes based on scintillators is therefore to be seized and the improvement of existing indirect detection technologies needs to be further investigated.

An indirect detector essentially combines two elements: a scintillator, which primarily converts incoming X-rays into multiple optical photons, and a two-dimensional photosensor (camera), which subsequently converts this scintillation light into an electric signal. The coupling of these two elements can be achieved with optics, which allows changing the magnification of the optical image. Alternatively, the scintillator can be directly coupled to the photosensors with the help of a fiber-optics plate. This rather dense assembly of optical fibers protects the photosensor from direct irradiation while preserving the quality of the transmitted optical image. With a transmission efficiency usually higher than 50%, such assembly provides a higher light-collection efficiency compared with a lens-coupling configuration. However, not all photosensors allow a direct coupling to a fiber-optics plate, depending on the architecture of the chip and possible system integration constraints (*e.g.* operating temperature, tolerance to a mechanical stress).

Early work reported single X-ray events detection with CCD cameras (Lumb & Holland, 1988 ▸; Gureyev *et al.*, 2001 ▸), with the limitation of requiring relatively long exposure times. Back-illuminated CCD technology significantly improved the performances (Mayo *et al.*, 2002 ▸; Miyata *et al.*, 2006 ▸). More recently, a group reported on the use of scintillators and scientific CMOS (sCMOS) cameras for such application (O’Connell *et al.*, 2020 ▸). These cameras offer fast acquisition rates and a very low readout noise. There is therefore a particular interest in developing versatile indirect detectors with high performances and single X-ray sensitivity at a reasonable cost.

The aim of this work is to investigate the performances of such systems based on state-of-the-art elements: a commercial sCMOS camera with fiber-optics plate coupling and a very common Gd_2_O_2_S:Tb powder scintillator (Popovici *et al.*, 2004 ▸). Such scintillating screens (referred to as GOS, Gadox or P43) can provide a large and uniform field of view, are particularly bright and are often selected as an economical solution. Here, we present a simple method for evaluation of the single X-ray photon detection limit and demonstrate the validity of this approach with the selected detector at the ESRF-BM05 beamline. The study combines experimental data and Monte Carlo simulations to discuss the performances with an emphasis on the detection efficiency. We also identify current limitations and prepare the ground for future R&D to be able to assemble a detector combining the advantages of direct and indirect detection schemes.

## 2. Material and methods

In this article, we use ‘ph’ and ‘phX’ to refer to a scintillation (optical) photon and an X-ray photon, respectively.

### 2.1. Camera parameters and calibration

The present study was performed with a sCMOS camera (model Zyla-HF from Andor) with a pixel size of 6.5 µm. The camera is equipped with a fiber-optics plate (45 mm long) allowing a direct coupling (in-path configuration with respect to the beam) of the scintillating screen. Table 1 ▸ provides the electronic gain *K*, the readout noise σ_d_ (expressed in r.m.s.), the absolute sensitivity μ_e.min_ [see equation (1)] and the saturation capacity μ_e.sat_ of the camera, measured according to the EMVA Standard 1288 (Jähne, 2010 ▸).

To obtain a detector sensitive to single X-ray photons, the readout noise is the key parameter. Other sources of noise such as dark-signal non-uniformities can indeed be compensated by acquiring dark reference images. At short exposures (<50 ms), the dark current can also safely be neglected.

**Figure 1:**
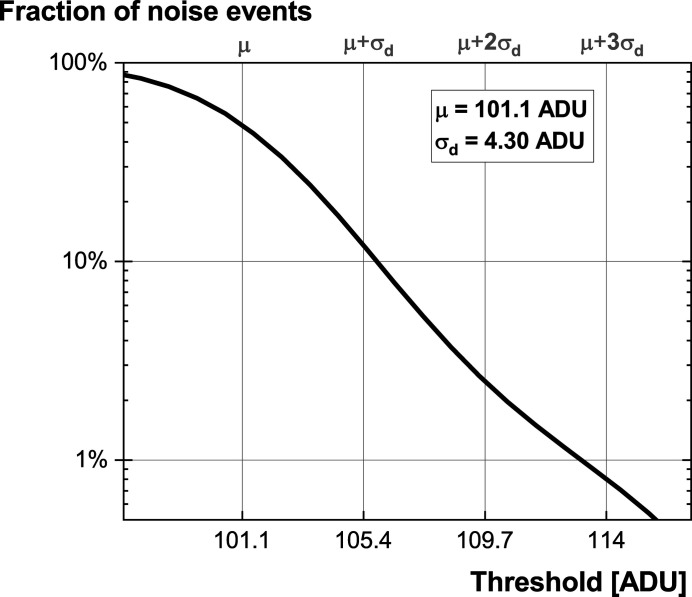
Noise distribution of the camera (operated in low noise mode) as a function of the applied threshold. The mean value μ and the standard deviation σ_d_ are provided as scaling factors.

The readout noise σ_d_ quantifies the level of noise but cannot directly be used as a threshold value for the separation of the events. To illustrate this, the distribution of noise events is provided in Fig. 1 ▸. To reject 99% of noise events, the threshold must be set at 113.1 ADU, which corresponds to ∼3.87 e, whereas σ_d_ was measured at 1.27 e.

In this work, the applied threshold is the camera absolute sensitivity μ_e.min_, as defined by Jähne (2010 ▸),

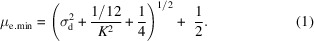

**Figure 2:**
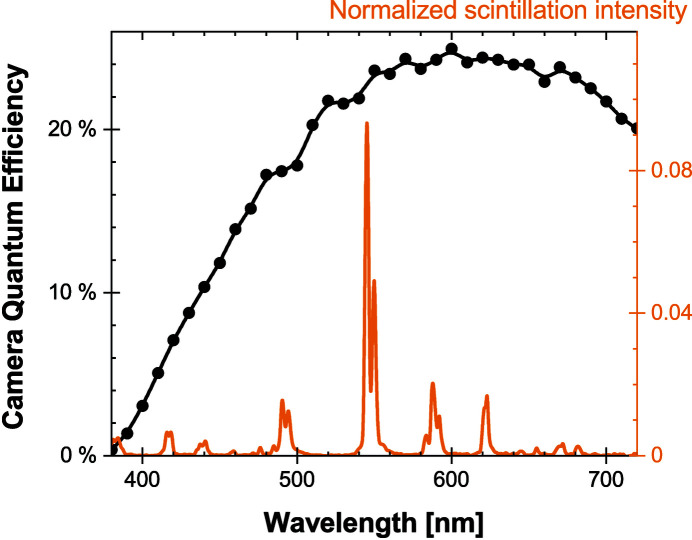
Quantum efficiency of the Zyla-HF camera (equipped with a fiber-optics plate) and an emission spectrum of a GOS:Tb scintillating screen. A convolution of both curves yields QE_sys_ = 21.0%.

The quantum efficiency of the camera was measured with monochromatic optical photons against a reference power meter. The data are provided in Fig. 2 ▸. The values are noticeably lower compared with the sCMOS sensor alone. This is attributed to the moderate transmission (∼40%) of the scintillation light through the fiber-optics plate. Losses are caused by the Fresnel reflections at the interfaces, the fill factor between the core of the fibers and their cladding, the density of absorbing fibers, and bulk absorption in the glass.

### 2.2. Light-output measurements with an X-ray generator

In order to estimate the light output of the scintillating screens and properly normalize the computed values, a uniform distribution of X-rays is needed. An X-ray generator (model XRG3D from Inel) operated at 25 kV and equipped with a molybdenum anode and a zirconium filter (100 µm thick) provides a rather narrow energy distribution. The flux was measured by direct conversion of the X-rays into a silicon photodiode (500 µm thick). For a given deposited energy *E*_dep_ onto a surface area *S*, the measured current *I* provides the incoming X-ray flux ϕ by 

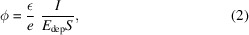

with ε = 3.66 eV and *e* = 1.602 × 10^−19^ C.

When the beam is non-monochromatic, the computation of the energy deposit is not straightforward and all contributing energies *E*_*i*_ of weight *w*_*i*_ must be taken into account, 

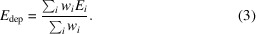

To determine accurately this energy deposit, the energy distribution of the generated X-rays was measured with a silicon drift detector (SDD) (model SXD30M-500 from Mirion) with a 500 µm-thick silicon sensor. A set of slits (150 µm × 150 µm) was located in front of the SDD window. The acquired spectrum is corrected by the absorption efficiency of the silicon layer, which is computed from the photon attenuation coefficient μ(*E*_*i*_) provided by Berger *et al.* (2010 ▸) and the sensor thickness *t*_SDD_,

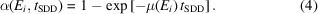

The corrected spectrum is then weighted by the absorption efficiency of the photodiode to compute the average energy deposit. Naturally, when the SDD and the photodiode are made of the same material and have the same sensor thickness, the correction of the spectrum is no longer needed and the weight factors *w*_*i*_ from equation (3) simply become the number of counts per bin from the acquired energy spectrum.

**Figure 3:**
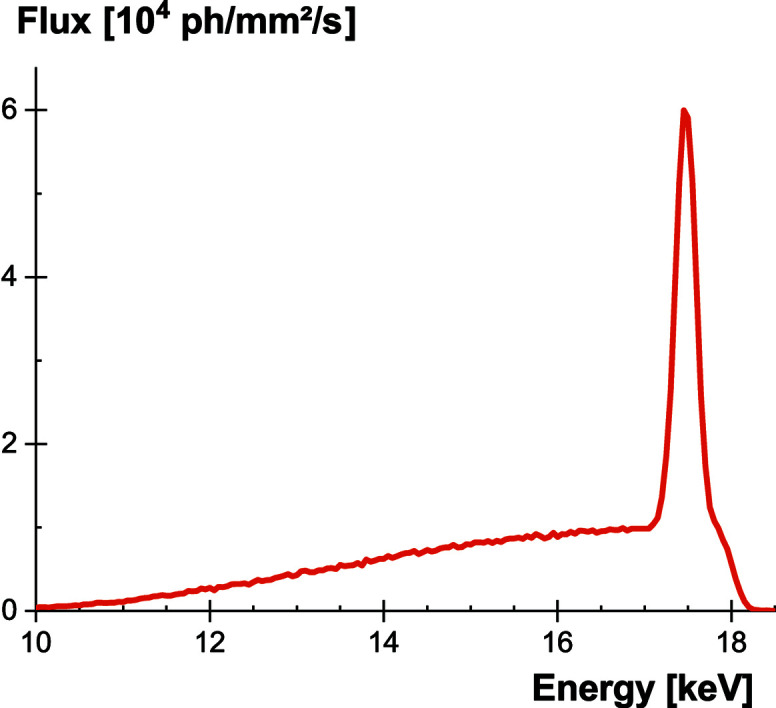
Flux and energy of generated X-rays with a molybdenum anode and a 100 µm zirconium filter, measured at a distance of 125 cm.

Combining the energy spectrum of the X-ray tube and the current measured by the photodiode provides the incoming X-ray flux, as displayed in Fig. 3 ▸. In our experimental conditions, the integrated flux is 1.10 × 10^6^ phX mm^−2^ s^−1^ and the average energy deposit is 15.4 keV. Careful measurements of these values are needed in order to be able to normalize meaningfully the light output of the scintillating screens.

### 2.3. Single X-ray detection at the ESRF-BM05 beamline

**Figure 4:**
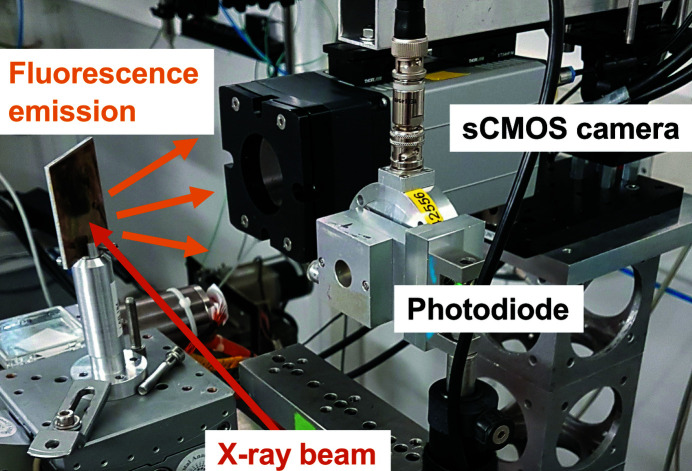
The experimental setup for measurement of single X-ray photons. The fluorescent target is oriented at 45° and the camera at 90°, with respect to the beam path.

To be able to evaluate the single-photon detection ability of our detector, a uniform monochromatic beam of relatively low intensity is required. Therefore, experiments were performed at the ESRF-BM05 beamline (Ziegler *et al.*, 2004 ▸), which allows scanning energies in the 10–80 keV range. A low X-ray fluence was first obtained by strongly attenuating the direct beam with 1 mm of tungsten. This approach turned out not to be the best solution since harmonic components (*e.g.* 3 × *E*_0_), which are not attenuated as efficiently, tend to dominate the filtered beam (beam-hardening effect). As a consequence, it becomes difficult to control the energy of the X-ray photons interacting in the scintillating screens. For this reason, the experiments were repeated with the beam centered on fluorescence foils oriented at 45°, while the camera was oriented perpendicular to the beam path, as shown in Fig. 4 ▸. The beam energy was adjusted to slightly exceed the *K*-edge energy, and the chosen orientation makes sure the fluorescence from the *K*α,*K*β transitions is the main contributor to the collected signal (*i.e.* neglecting the direct beam and scattered X-rays). The transition energies for the various targets are provided in Table 2 ▸.

### 2.4. Scintillating screens

The scintillating screens selected for this study are based on a mixture of scintillating powder (G_2_O_2_S:Tb, average grain size of 2.5 µm) and a polymer binder (acrylate) deposited on a reflective coating (Mylar foil, thickness of 50 µm). The weight fraction of the powder is estimated at 85%. Such gadolinium oxide screens, often referred to as Gadox or P43, are very common in medical and synchrotron applications. They are easy to produce, even in large dimensions, and are very competitive in terms of both performance and price.

The emission spectra of the scintillating screens were acquired with a monochromator (model 77200 from Oriel) and a photomultiplier tube (model R3896 from Hamamatsu). A typical spectrum acquired with an average X-ray energy of 15.4 keV is displayed in Fig. 2 ▸. The quantum efficiency of the system (QE_sys_) is obtained by combining the emission spectrum of the scintillating screen with the quantum efficiency of the camera (combining efficiencies of the photosensor and the fiber optics). This defines the probability to detect a scintillation photon. For the detecting system combining the scintillating screens and the Zyla-HF camera, we obtain QE_sys_ = 21.0%.

The density of our scintillating screens was determined from precise measurements of their weight (HR-150AZ balance from A&D Weighing) and appears to be slightly varying with the screen thickness, as shown in Table 3 ▸. The absorption efficiency defined in equation (4) can be used to approximate the energy transferred to the scintillating layer. The approximation is valid when the energy of the X-rays is below the scintillator *K*-edge energies to be able to neglect the production of fluorescence X-rays. This is of special importance since the considered scintillators are rather thin and the escape of the fluorescence X-rays will lower the effective energy deposit in that energy range.

**Figure 5:**
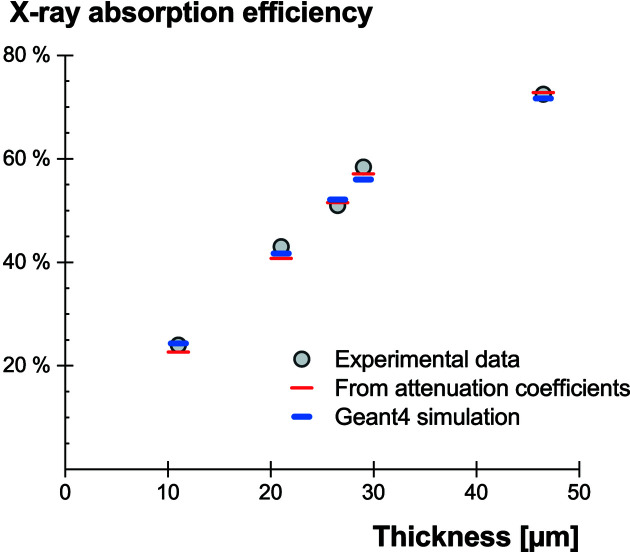
Absorption efficiencies at 15.4 keV from experimental data, compared with values computed from the X-ray attenuation coefficients and from *Geant4* simulation.

The GOS:Tb screens were inserted in the path of the X-ray beam. The camera was coupled to a dense scintillator (micro-columnar CsI, thickness of 230 µm) and images were taken with the X-ray beam partially blocked by the GOS:Tb screens. Comparing the collected signal in both covered and uncovered regions then provides a measurement of the X-ray absorption efficiency of the GOS:Tb screens. The acquired data (provided in Table 3 ▸) are in good agreement with the expected values, computed from the X-ray generator energy spectrum and flux, as illustrated in Fig. 5 ▸.

### 2.5. *Geant4* simulations

In some experimental conditions, for example when approaching the *K*-edge energies, Monte Carlo simulations provide a better estimate of the effective energy deposit. Simulations were performed for this purpose with the *Geant4* toolkit (Agostinelli *et al.*, 2003 ▸). For simplicity, individual powder grains were not modeled and a mixture of G_2_O_2_S:Tb and acrylic materials was considered as the scintillating layer. This assumption is expected to be valid when excluding the photon transport from the simulations. The modeled composition, weight fractions and geometrical dimensions are identical to those described in Section 2.4. The simulations were run with the low-energy Livermore physics model (Apostolakis *et al.*, 1999 ▸; Ivanchenko *et al.*, 2011 ▸) and the number of X-ray interactions was chosen to obtain a statistical error below 1%. The X-ray absorption efficiencies computed from the simulations were added to Fig. 5 ▸ and a good agreement is again observed.

### 2.6. Spatial resolution

To evaluate the spatial resolution of the system, the slanted-edge method was applied (Zhu *et al.*, 1995 ▸; Samei *et al.*, 1998 ▸). A tungsten edge (1 mm thick) was carefully aligned with the scintillator plane. The edge was rotated by ∼8° with respect to the camera rows to avoid a favored orientation of the pixels. A set of edge images was acquired at half the sensor dynamic range.

## 3. Lower limit in X-ray detection

The driving criterion to design a detector sensitive to single X-ray photons is to separate the signal generated upon conversion of the X-ray photon from the noise of the system. The entire conversion process must therefore be considered.

### 3.1. Light output

**Figure 6:**
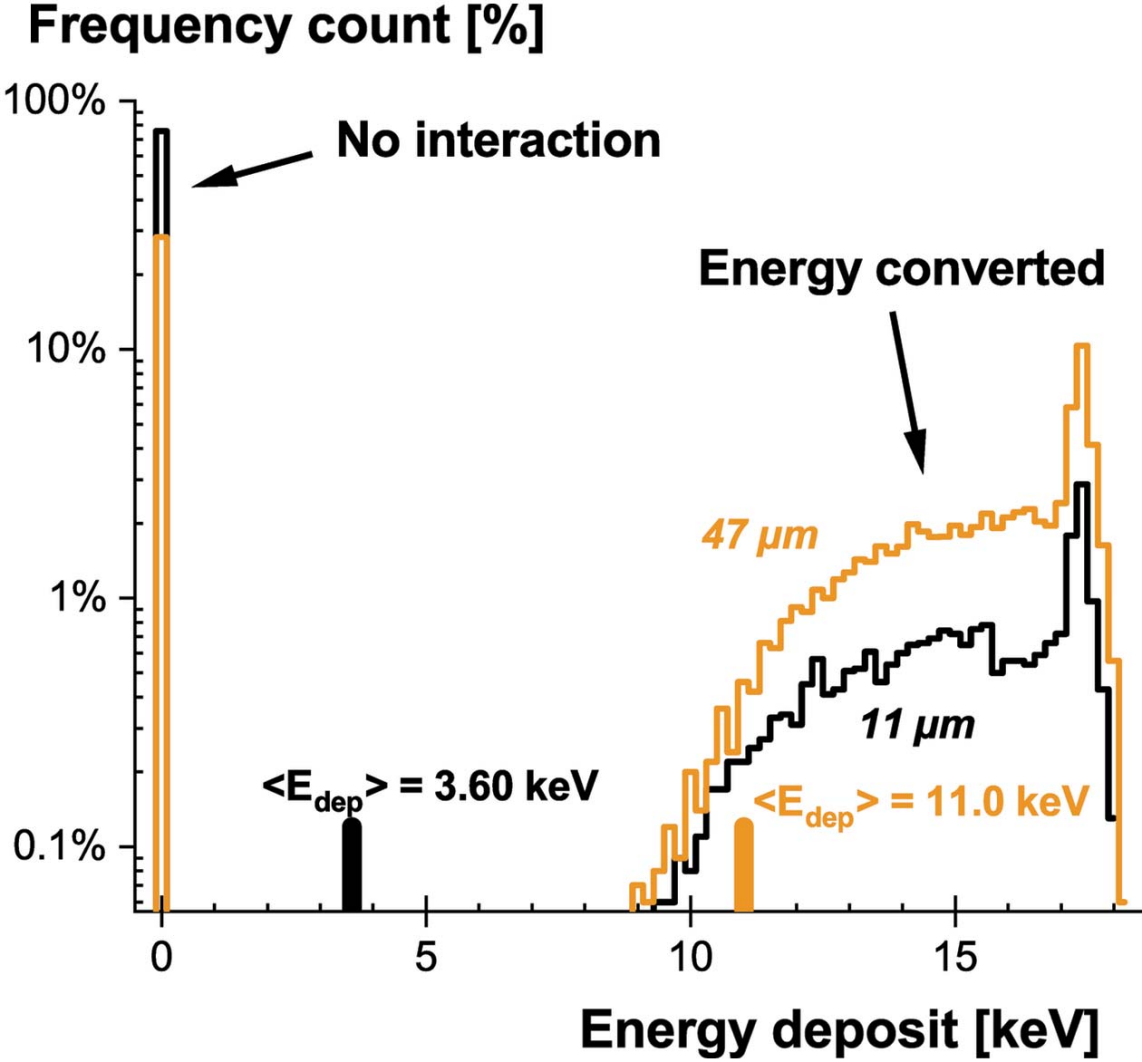
*Geant4* simulation (10000 events) of the energy deposit in GOS:Tb scintillators of thicknesses 11 and 47 µm. The corresponding average energy deposits (〈*E*_dep_〉) are also provided.

Based on the calibration of our system presented in Section 2, a light-output measurement can provide a quick assessment of the minimum energy required to obtain signals distinguishable from the camera noise. Flat-field images were acquired under uniform illumination, corrected for the dark signal and carefully normalized by the average energy deposited in the scintillating screens, computed from the absorption efficiency and the energy spectra from the X-ray generator [equation (3)]. To better visualize the meaning of this average energy, the histogram of the energy deposit is provided in Fig. 6 ▸. Due to the limited thickness, a considerable fraction of X-ray photons do not interact in the scintillator. Additionally, a fluctuation due to the energy distribution of the generated X-rays is observed for those that convert their energy. When collecting the signal over a large number of events (*e.g.* acquisition without threshold to generate images exploiting the dynamic range of the sensor), the normalization requires computing the average value of this statistical distribution. The computed values are provided in Fig. 6 ▸ for illustration. Applying a threshold in energy would definitely help in separating events and easily increase the quality of the recorded data.

The signal collected by the camera, the average energy deposit and the computed light output are provided in Table 4 ▸. The GOS:Tb screens have a light output in the range 30–34 ph keV^−1^. This value slightly depends on the screen thickness and translates to a light-collection efficiency of 44–52% with respect to the intrinsic light yield (Wickersheim *et al.*, 1970 ▸; Ludwig, 1971 ▸). Higher light-output values were reported earlier (Howansky *et al.*, 2018 ▸; Dow *et al.*, 2021 ▸) but the GOS:Tb screens differ in thickness, grain size and assembly. This may have an effect on the estimated single-photon sensitivity thresholds but also leaves room for further improvement.

### 3.2. Light sharing

**Figure 7:**
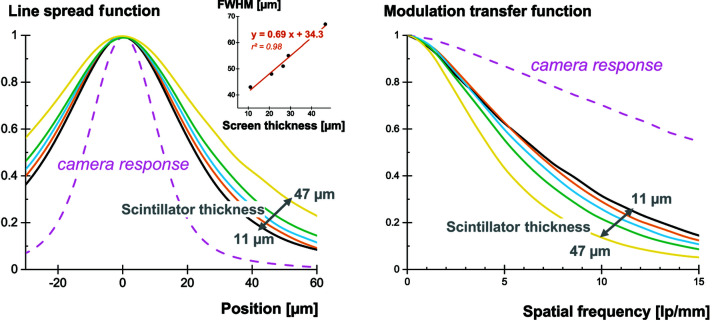
LSF, FWHM (inset) and MTF of the GOS:Tb scintillating screens. The system response of the camera is provided for information.

The scintillation light generated upon conversion of the X-ray photons is emitted isotropically. The light output measured earlier is therefore dependent on the detecting system and especially on the light-collection mechanisms. Changing the length of the fiber-optics plate, the density of absorbing fibers, the quality of their optical transmission will for instance affect the way the scintillation light is collected. Additionally, the scintillation light is usually distributed over a large number of pixels. This spreading of the scintillation light can be quantified by measuring the spatial resolution of the detecting system. The line-spread function (LSF) and the modulation transfer function (MTF) were measured according to the methods described in Section 2.6. The results are displayed in Fig. 7 ▸. The full width at half-maximum (FWHM) of the LSF does not scale down to zero for very thin layers. A minimum of 35 µm seems to be the limit for our system. This does not seem to correspond to the camera resolution limit, as demonstrated by the MTF measurement of the camera itself. It is more likely to be caused by the large numerical aperture (NA = 1) of the fiber-optics plate coupled to the camera and the large amount of scattering involved in the light propagation in powder-based scintillators.

**Figure 8:**
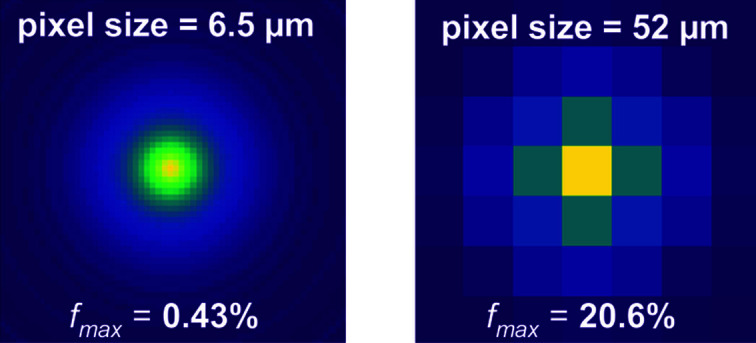
Sharing of the scintillation light over the camera pixels (6.5 and 52 µm) with a 47 µm-thick GOS:Tb screen. The corresponding fraction of scintillation light *f*_max_ collected by the brightest pixel is also provided.

The LSF allows predicting of the spreading of the scintillation light on the camera pixels. It is essential to quantify this parameter, since at low energy only a few pixels will collect enough light to generate a signal sufficiently higher than the noise. In Fig. 8 ▸ we illustrate how the signal is distributed over pixels of 6.5 and 52 µm (*e.g.* with 8 × 8 pixel binning) in the case of a 47 µm-thick GOS:Tb screen. The central (brightest) pixel collects 0.4% and 20%, respectively. Naturally, a larger pixel also comes with a higher amount of noise, but the scaling is different and an optimum can be found.

**Figure 9:**
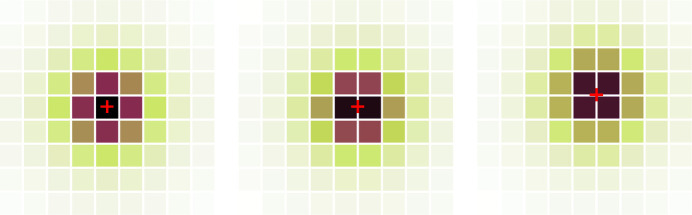
Three configurations of light sharing depending on the location of the X-ray interaction point with respect to the pixel matrix: center, edge or corner of a pixel.

In practice, the light distribution is unlikely to be in coincidence with the center of the camera pixels. Deviating from this position shares the maximum of the light distribution among closest neighbors. In Fig. 9 ▸, we illustrate this with three typical configurations: distribution aligned with the center, one edge and one corner of a pixel.

### 3.3. Detection limit

Based on the normalized light output, the light-spreading characteristics and the camera noise, we can now define a lower boundary for the detector. The detection threshold must be set in a way that minimizes the contribution of noise events. In that sense, containing all the scintillation light in one pixel does not necessarily benefit the single X-ray detection limit as isolated bright pixels can indeed be hard to discriminate from noise events (*e.g.* ‘hot pixels’, cosmic events, direct conversion of X-rays). In a conservative approach, we decide here to consider a simple system with no particular discrimination abilities. And in order to separate single X-ray events from the noise, we define the single X-ray detection threshold as the following condition: at least four neighboring pixels have a signal above the absolute sensitivity threshold of the camera. To test this condition, we rank the signals *A*_*i*_ collected by *n* pixels in descending order and we normalize the distribution to its sum,

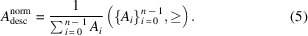

The lowest fraction of signal collected in a cluster of four pixels then is 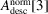 . As already indicated in Fig. 9 ▸, this value depends on the location (*x*, *y*) of the X-ray interaction. The situation is favorable when the light distribution is centered at a pixel corner and unfavorable when slightly off from the center of the pixel. To obtain the fine structure of this dependence on the interaction point, we can compute a pixel response map *S*_pixel_ with a set of (*x*, *y*) coordinates distributed over the pixel area,

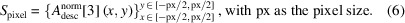

**Figure 10:**
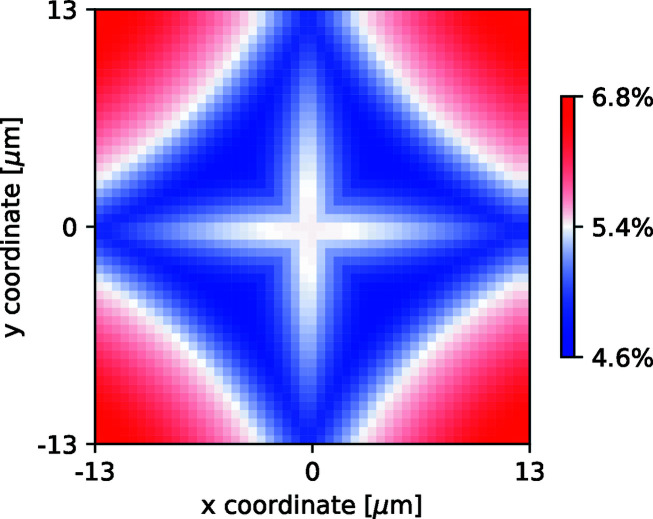
A map of the fraction of light collected (*S*_pixel_) depending on the X-rays interaction point for a 29 µm-thick GOS:Tb screen and a pixel size of 26 µm. Over the pixel area, the average fraction of light collected is 5.4%, whereas the minimum fraction of signal collected (η_min_) is 4.6%. The center of the pixel is located at [0,0].

An example of such a map is displayed in Fig. 10 ▸. In a conservative approach, we would like to have all interactions yielding a signal above the single-photon threshold. This means that only the lowest value of the pixel response map is considered. The minimum light collection over the pixel surface is thus defined by

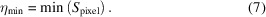

In the definition of η_min_, the condition of contiguity of the four brightest pixels is not tested. However, because of the symmetry of the point-spread function and the absence of noise events in the modeling, the highest signals are necessarily collected from neighboring pixels. The algorithm applied on real data should of course include this condition to properly exclude background events.

We now have all the elements in hand to define the single X-ray photon sensitivity threshold. For a given X-ray energy, we can predict how much light will be converted by the scintillator, how this light will be collected and shared among the camera pixels, and how the collected signal compares with the noise level. In Table 5 ▸, we provide the single-photon thresholds for the GOS:Tb screens studied here and the native pixel size of the camera (6.5 µm). The computed thresholds correspond to the lowest X-ray energy that allows single-photon detection. Only the very thin screens (<20 µm) approach the 30 keV threshold.

**Figure 11:**
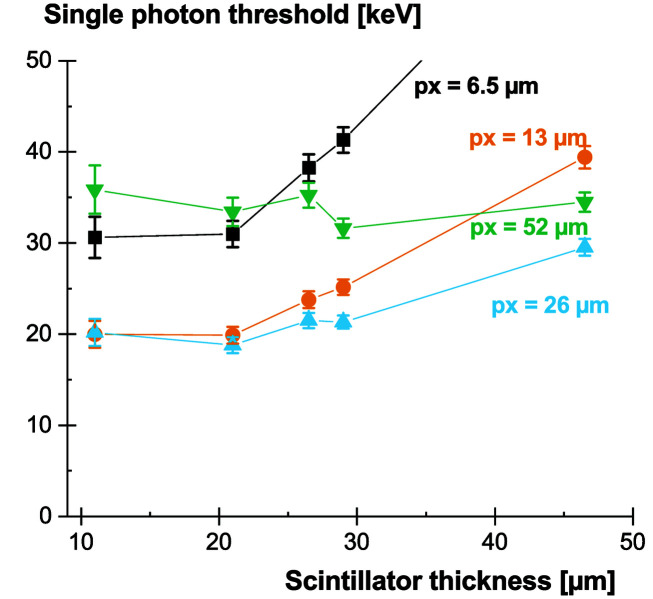
Single X-ray detection thresholds for GOS:Tb scintillating screens as a function of screen thickness and camera pixel size (px).

We can also investigate the influence of the pixel size by computing the pixel response map with different pixel binning. The noise level scales with the square root of the pixel area. The light fraction scales linearly with the pixel area, within the limits of the LSF. In Fig. 11 ▸, we display the achievable single-photon thresholds as a function of pixel size and screen thickness.

A good compromise is reached with a pixel size of 26 µm (binning 4 × 4). The computed values are reported in Table 6 ▸. In terms of screen thicknesses, the best option is to select the thickest screen allowing the considered single-photon threshold (*e.g.* 29 µm in this case). This enables one to increase the absorption efficiency while keeping a similar sensitivity. However, the major limitation of the scintillating screens considered here is the lack of an efficient spatial containment of the scintillation light. Because of this, rather thin screens have to be selected. Micro-structured scintillating screens (Svenonius *et al.*, 2009 ▸; Sahlholm *et al.*, 2011 ▸; Chen *et al.*, 2018 ▸) offer interesting perspectives to further improve the compromise between absorption efficiency and single-photon sensitivity.

As mentioned earlier, the defined single X-ray detection threshold is rather conservative and the computed thresholds can in practice be lowered if the detecting system is based on optimized technologies.

## 4. Proof of concept

**Figure 12:**
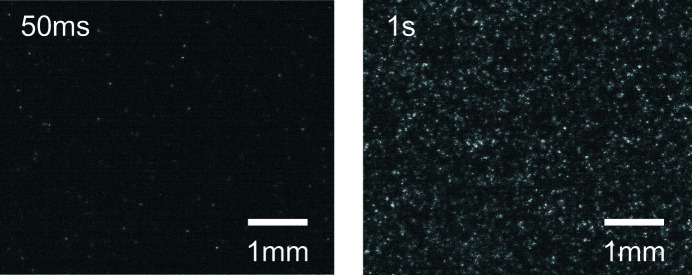
Unprocessed images acquired with a tungsten target (the energy range is 58–69 keV) at two exposure times. The scintillator is an 11 µm GOS:Tb screen and the pixel size is 6.5 µm.

In order to confirm the minimum energy required to achieve single X-ray photon detection, we collected experimental data at the ESRF-BM05 beamline with X-ray energies from 8 to 50 keV. As described in Section 2.3, the scintillating screens were exposed to the direct beam with a strong attenuation or to the fluorescence from various targets. At the maximum energy range, single X-ray photons become easily identifiable, even without background subtraction, see Fig. 12 ▸.

**Figure 13:**
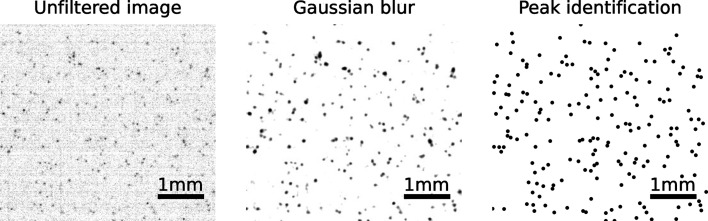
A background-subtracted image (left), a Gaussian filtered image (middle) and the location of identified X-ray photons (right). For better visibility, the colorscale is inverted. The X-ray energy is 40 keV, the scintillator is an 11 µm GOS:Tb screen and the pixel size is 6.5 µm.

When approaching the detection limit, the separation of the X-ray photons is more difficult. Therefore, we decided to apply a Gaussian filter (σ = 3) to the background-corrected images. As shown in Fig. 13 ▸, this technique conveniently highlights single X-ray photons. Subsequently, to ease the photon counting, a peak-identification algorithm based on amplitude and cluster size was applied: a Python code based on an inverse watershed from the *pyFAI* package (Kieffer & Ashiotis, 2014 ▸).

**Figure 14:**
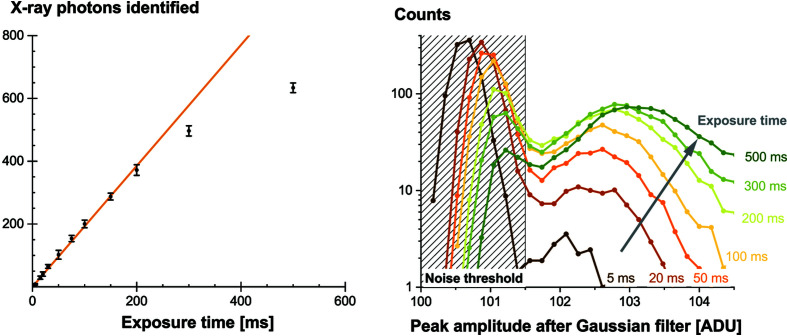
Linearity of the number of identified photons as a function of the exposure time (left) and influence on the identified peak amplitude (right). The X-ray energy is 40 keV, the scintillator is an 11 µm GOS:Tb powder screen and the pixel size is 6.5 µm.

This simple data-processing method was proven to be robust in highlighting single X-ray events. Fig. 14 ▸ illustrates this by comparing the number of X-ray photons identified at increasing exposure times. A deviation from the linearity is observed starting from ∼400 events distributed on the acquired image (21.5 mm^2^). The amplitude of the detected peaks varies slightly with the number of interacting X-ray photons. Two reasons can explain this. First, the applied threshold does not strictly exclude noise events. And since the contribution from the noise becomes higher when only a few X-rays interact, this tends to move the observed peak in the histogram of Fig. 14 ▸ to lower amplitudes. Second, when the number of interacting X-rays is increasing, the overlap of the point-spread functions becomes more important. This means that the amplitude of the detected peaks is higher since multiple peaks contribute to it.

Because of the applied filtering, the amplitude values presented here (although expressed in ADU) cannot directly be compared with the values from Section 3. As shown in Fig. 13 ▸, the noise threshold can actually be reduced from 6.3 to 1.5 ADU. Naturally, the collected signal also appears lower because of the applied Gaussian blur. This difference in signal amplitude does not prevent the identification of the single X-ray detection limit since this value is an energy threshold, expressed in keV.

**Figure 15:**
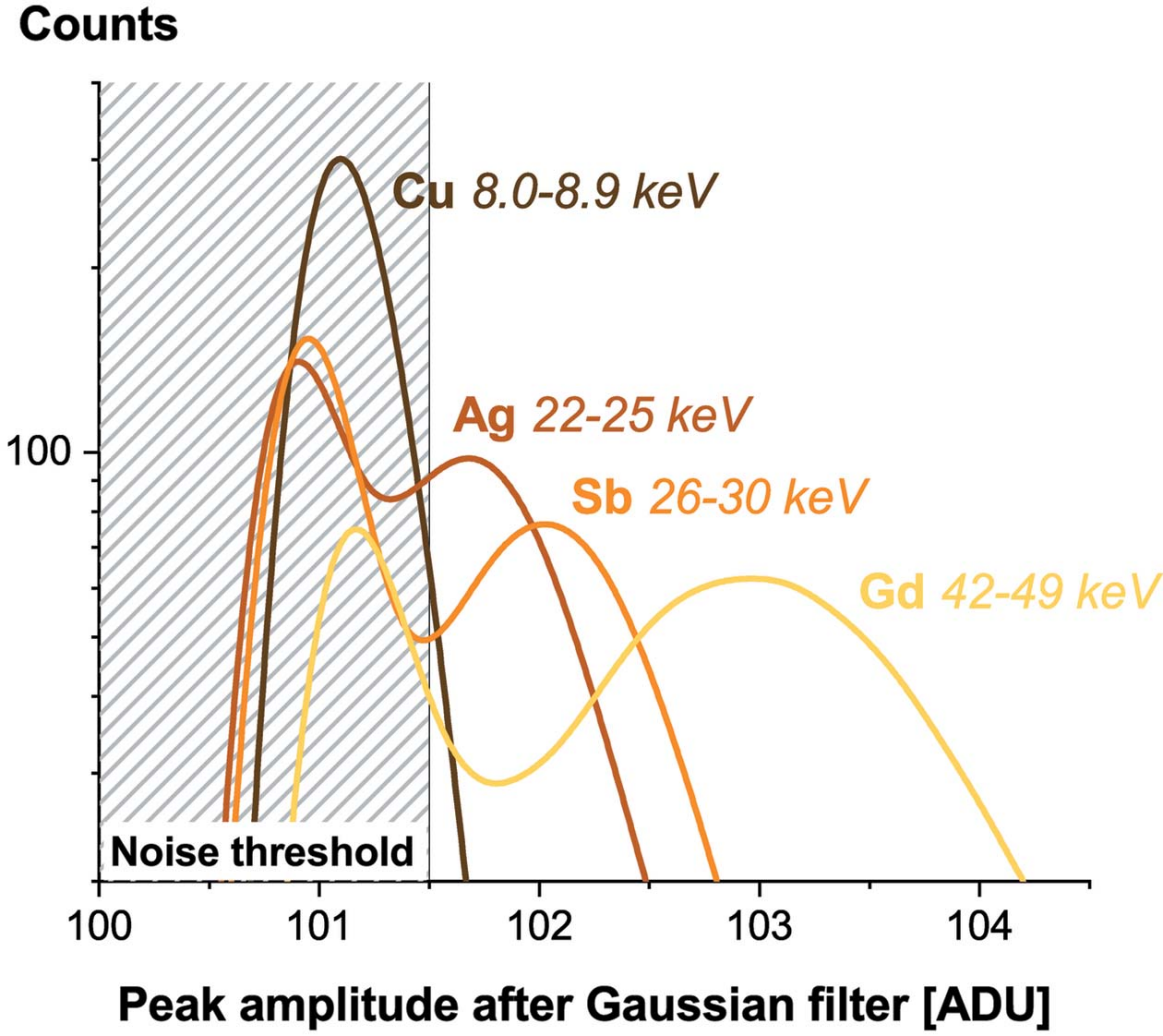
A histogram of detected peak amplitudes for various fluorescence targets. The scintillator is an 11 µm GOS:Tb screen and the pixel size is 6.5 µm.

In order to obtain a clean signal that excludes possible harmonics in the monochromated energy, fluorescent targets were used and peak-amplitude histograms were recorded at various energies. The results are shown in Fig. 15 ▸. While the Cu target does not allow any identification of single X-ray photons, a clear separation from the noise is observed with the Sb target. The corresponding energy range is 26–30 keV, which is consistent with the numbers announced in Section 3.

## 5. Discussion

The experimental demonstration of the ability to detect single X-ray photons confirmed the results predicted from careful characterization of the light output of the scintillators and the performances of the camera. This allows setting up of a screening campaign of the available scintillators and conveniently determining their suitability for this application. This validated approach is rather simple to set up and requires limited equipment. The critical aspect is, in fact, to carefully characterize the system beforehand.

More generally, the fact that single X-ray photons can readily be measured with commercial systems equipped with widely distributed scintillating screens gives confidence in the possibility to further optimize the system discussed here. The results help in defining the current threshold and the various parameters, which can help improve the performance. Prior work has already provided a carefully observation of single events with the objective to better understand the spreading of the scintillation light and its fluctuations (Howansky *et al.*, 2018 ▸). This work was based on an EM-CCD camera, which provides intense single X-ray photon signals. In our work, we propose to use a commercial sCMOS camera coupled to a fiber-optics plate. This solution comes at a reasonable cost, which can be a decisive advantage with respect to other existing solutions. The simple method presented here for identifying the location of single X-ray photon interactions can be further improved. The chosen algorithm is not particularly optimized and leaves room for improvement to exceed the announced performances. More efficient approaches are, for instance, discussed by O’Connell *et al.* (2020 ▸). Such charge-cloud localization techniques could be applied offline, once the data are acquired, or, more interestingly, either during data collection (*e.g.* post-processing data pipeline) or directly in the field-programmable gate array (FPGA) programming of the sensor.

### 5.1. Improving the signal-to-noise ratio

One approach to further improve the performances of the system is to increase the signal-to-noise ratio. This can be achieved by selecting a better optimized camera. The distribution of the readout noise is of particular importance. A better containment of the pixel-to-pixel readout noise variations would be beneficial since the overall threshold could be lowered accordingly. Recently developed cameras (*e.g.* Orca-Quest from Hamamatsu) offer a readout below 1 e s^−1^, which paves the way to single optical photon detection. This is obviously of high interest for the applications discussed here. The overall noise of the system can indeed drastically be reduced by applying a threshold directly on the photosensor and thereby efficiently rejecting noise events. A good uniformity of both the sensor and the scintillator is to be maintained and therefore non-uniformities in dark signals and pixel response should be taken into account. As a general rule, reducing these non-uniformities helps in discriminating the events more accurately.

Another line of work is the quantum efficiency of the sensor. Back-illuminated sensors could be of interest since they can easily lower the single X-ray photon threshold by the gain in photodetection efficiency. All the available options do not however necessarily translate into efficient X-ray detectors. The necessity to couple the sensor to a fiber-optics plate sets strong constraints in terms of integration. The selection of the adequate fiber-optics plate (*e.g.* compatible with sensor cooling requirements and radiation hard) should not be overlooked. Furthermore, the quality of such coupling could in principle be further improved since more than half of the light emitted by the scintillator is currently lost during collection and transport in this optical element.

Another option is the direct coupling or deposition of the scintillator onto the photosensor. The scintillator must however be dense and thick enough to minimize direct interactions of X-ray photons into the photosensor, which otherwise compete with the conversion into the scintillator.

### 5.2. Designing fast systems

**Figure 16:**
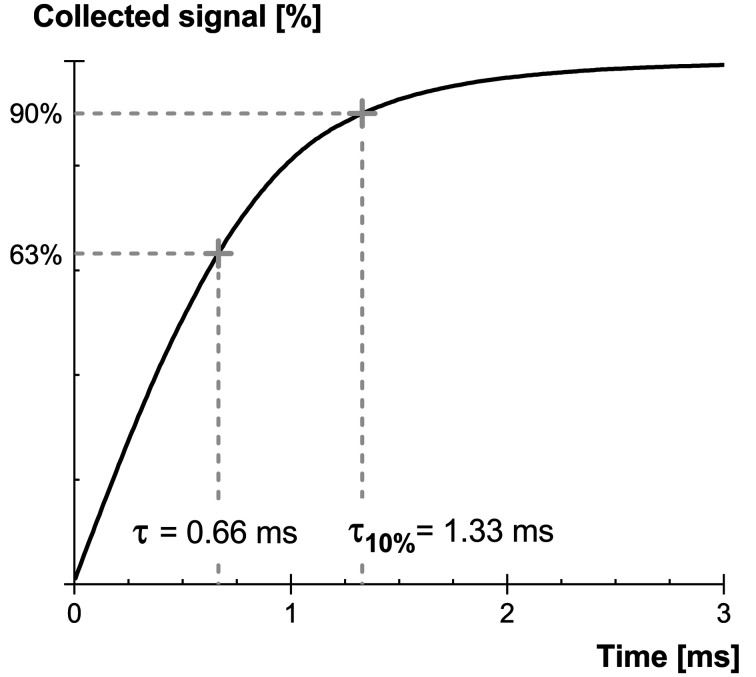
Collected signal as a function of the integration time for GOS:Tb screens.

Single X-ray photon detection with scintillators finds interest in many synchrotron applications. Two operating modes can be considered. The first one could be a hybrid system combining the advantages of integrating and photon-counting systems. In this case, the single-photon detection basically extends the dynamic range of the detector. The second operating mode would be to only consider single-photon events in the captured frames. This obviously supposes to achieve very short exposure times in order to adequately dilute the arriving X-ray photons on multiple frames when operating at very high flux. Exposure times in the tens of microseconds are easily achievable with recent sCMOS cameras. The limitation in this case is the scintillating screen. As shown in Fig. 16 ▸, the GOS:Tb screens investigated in this study limit the exposure time to a few milliseconds in order to maintain a sufficient integration of the scintillation light. Alternative scintillators (*e.g.* GOS:Pr) must therefore be considered instead, at the price of a possible loss in light output (Lecoq, 2016 ▸). Moreover, when considering alternative scintillating materials it is important to keep in mind the other requirements for an X-ray detector operating under high flux, in particular its radiation hardness.

To maintain decent acquisition times and fully exploit the brilliance of modern synchrotron light sources, the frame rate of the camera must be sufficiently high. The Zyla-HF camera selected for this study is limited to 100 frames per second. The scintillator itself could cope with up to 800 acquisitions per second without pile-up of the scintillation. The development of large-area photosensors with fast acquisition rates is therefore of particular interest. The CITIUS detector currently under development at SPring-8 (Hatsui, 2020 ▸) is able to operate at 17.4 kHz and can offer a field of view as high as 300 mm × 300 mm. The integration of fiber-optics plates in such detectors and the coupling to fast and efficient scintillators will truly allow a breakthrough in the performances of indirect detectors with single X-ray photon detection capabilities. This, of course, also requires designing an optimized data pipeline to cope with the demanding computational requirements to allow single-photon identification at very high frame rates.

### 5.3. X-ray stopping power and detection efficiency

The ability to detect single X-ray photons, although very attractive, should not be decoupled from the efficiency of such systems. Basically, only X-ray photons interacting in the material will generate useful signals and so the probability of interaction must be considered. If only a few events interact with the scintillator, additional noise caused by the poor photon statistics will degrade the quality of the acquired image. In principle, this source of noise could be suppressed if thresholds are properly applied during data acquisition. More events will be needed, due to the poor X-ray interaction probability, but they would not accumulate additional noise. Although, this reasoning is not valid for applications requiring minimal X-ray doses and single-shot imaging with no possibility to repeat the experiment.

To investigate this crucial aspect of the X-ray detection, additional *Geant4* simulations were performed. The energy deposited inside the scintillating layer, which we assume directly converts into scintillation light, was studied as a function of the X-ray beam energy. The scintillator contains gadolinium which causes fluorescence in the energy range considered. Due to the scintillator dimensions, these secondary X-rays are very likely to escape the initial inter­action point. They can deposit their energy further away, thus causing interactions unrelated to the impact point, which translate into a background noise. And, most probably, they do not interact at all in the scintillating layer, thereby causing partial energy deposits (visible as escape peaks in the peak amplitude spectrum). Since our study aims at exploiting the useful signals occurring during X-ray interactions, we only consider, in our simulations, a restricted volume corresponding to 50 µm × 50 µm × 50 µm. This assumption is conservative; if this simulated volume turns out to be too small then only part of the energy deposit is estimated by the simulations and the efficiency could in practice be higher than the predictions.

**Figure 17:**
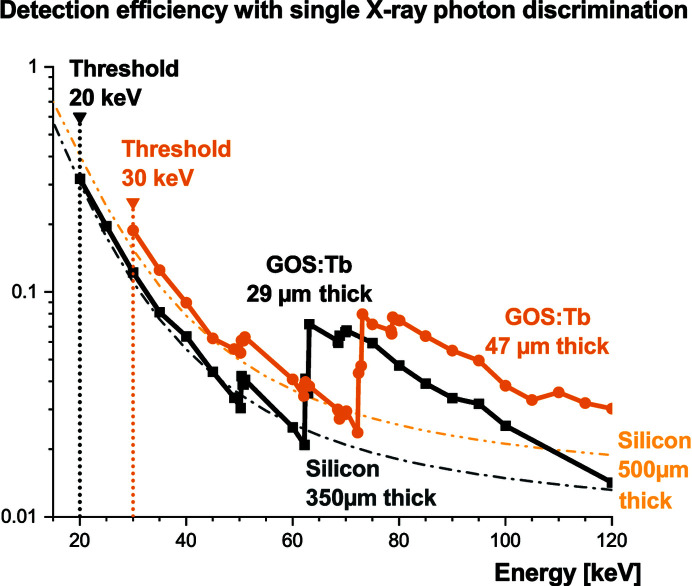
Detection efficiency with single X-ray photon discrimination for GOS:Tb powder screens based on *Geant4* simulation (10000 events per energy). To exclude out-of-pixel interactions due to fluorescence from the gadolinium as well as Compton scattering, the interaction region is limited to 50 µm × 50 µm. According to Section 3.3, the applied energy thresholds allow single X-ray photons to be detected with our detection scheme. The absorption efficiencies of silicon-based direct detectors (Berger *et al.*, 2010 ▸) are provided for comparison.

Fig. 17 ▸ displays the results of these simulations. In the 20–60 keV range, the efficiencies of the GOS:Tb screens in our detection scheme turn out to be substantially similar to that of silicon-based direct detectors. This, in itself, is of interest since the detection system discussed here can be assembled at a reasonable cost and with an excellent uniformity. The radiation hardness of scintillating screens also makes them more robust for in-beam measurements. Besides, the assembly with a fiber-optics plate makes it convenient to exchange the scintillating screen in case of extensive damage.

Above 60 keV, the gain in efficiency with respect to silicon is significant. The simulated efficiency is lower with respect to a naive computation based on the material cross section. As explained earlier, we decided here to consider the effective energy deposit (*i.e.* the energy absorbed and transferred to the scintillating layer), instead of only the X-ray absorption efficiency, since it is more likely to reflect the true response of the detector.

**Figure 18:**
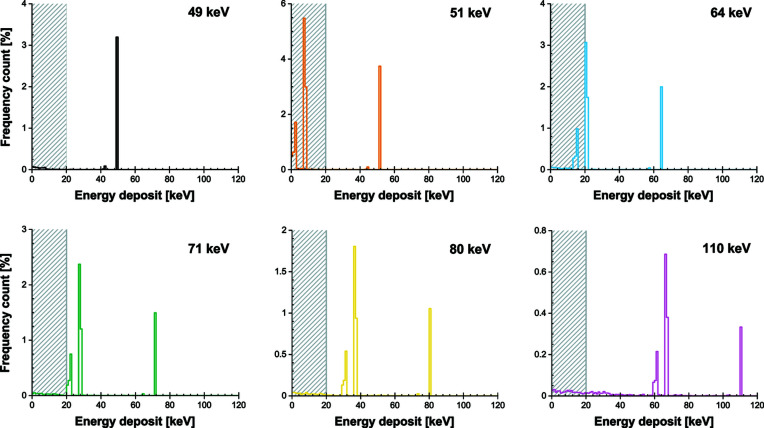
Distribution of energy deposits as a function of X-ray energy for 29 µm-thick GOS:Tb based on *Geant4* simulations (100000 events). To exclude out-of-pixel interactions due to fluorescence from the gadolinium as well as Compton scattering, the interaction region is limited to 50 µm × 50 µm.

We also observe additional edges in the efficiency curves and they appear to be shifted with respect to the *K*-edge of the gadolinium (50.207 keV). As depicted in Fig. 18 ▸, this is caused by the threshold applied to ensure single X-ray photon detection with our detecting scheme. Just above the *K*-edge energy, the escape peaks are visible in the histogram but they are discarded by the energy threshold *E*_THL_. These escape peaks only start to contribute to the detector efficiency when the X-ray beam energy is higher than *E*_*K*_ + *E*_THL_, where *E*_*K*_ is the *K*-series energies of gadolinium provided in Table 2 ▸. The first emission line is thus observed at 62.309 keV and 72.309 keV for 29 µm and 47 µm screens, respectively.

With an X-ray beam above 80 keV, interactions based on Compton scattering are contributing to the conversion process. This slightly increases the detection efficiency but comes at the price of an added background on surrounding pixels. More generally, Fig. 18 ▸ also highlights the fact that the energy-conversion mechanisms do not systematically yield a deposited energy equal to the beam energy. This observation is important since the threshold therefore needs to be kept at its minimal value to ensure the maximum detector efficiency. The fact that events with the same incoming energy can generate a fluctuating detector response is usually problematic. The so-called Swank noise (Swank, 1973 ▸) and Lubberts effect (Lubberts, 1968 ▸) for scintillating screens quantify this effect very well. However, it is possible with a detector sensitive to single X-ray photons to limit its impact. This is the advantage of a photon-counting approach, which translates the energy conversion into binary information (interaction or no interaction).

Finally, the simulated energy deposits discussed here are a simplified model. They do not entirely reflect the complexity of the energy-conversion processes occurring in scintillating materials. In particular, the fluctuations caused by the scintillation-light generation, propagation and extraction are overlooked. The clean histograms provided in Fig. 18 ▸ will translate poorly when taking into account the optical photon statistics. This, in turn, is likely to translate to a more difficult application of the threshold, and possibly at the cost of a lower detector efficiency.

In order to deepen the understanding of the detector and also study the spatial dependence of the signal-to-noise ratio, measuring the detective quantum efficiency is of high interest. It quantifies the sensitivity of the system, taking into account all the processes. It also provides an excellent figure of merit to compare systems of different technology. However, this study goes slightly beyond the scope of this contribution. Future work will study a wider set of scintillating screens and compare this parameter with the efficiencies predicted by our simulations.

### 5.4. Impact on spatial resolution

**Figure 19:**
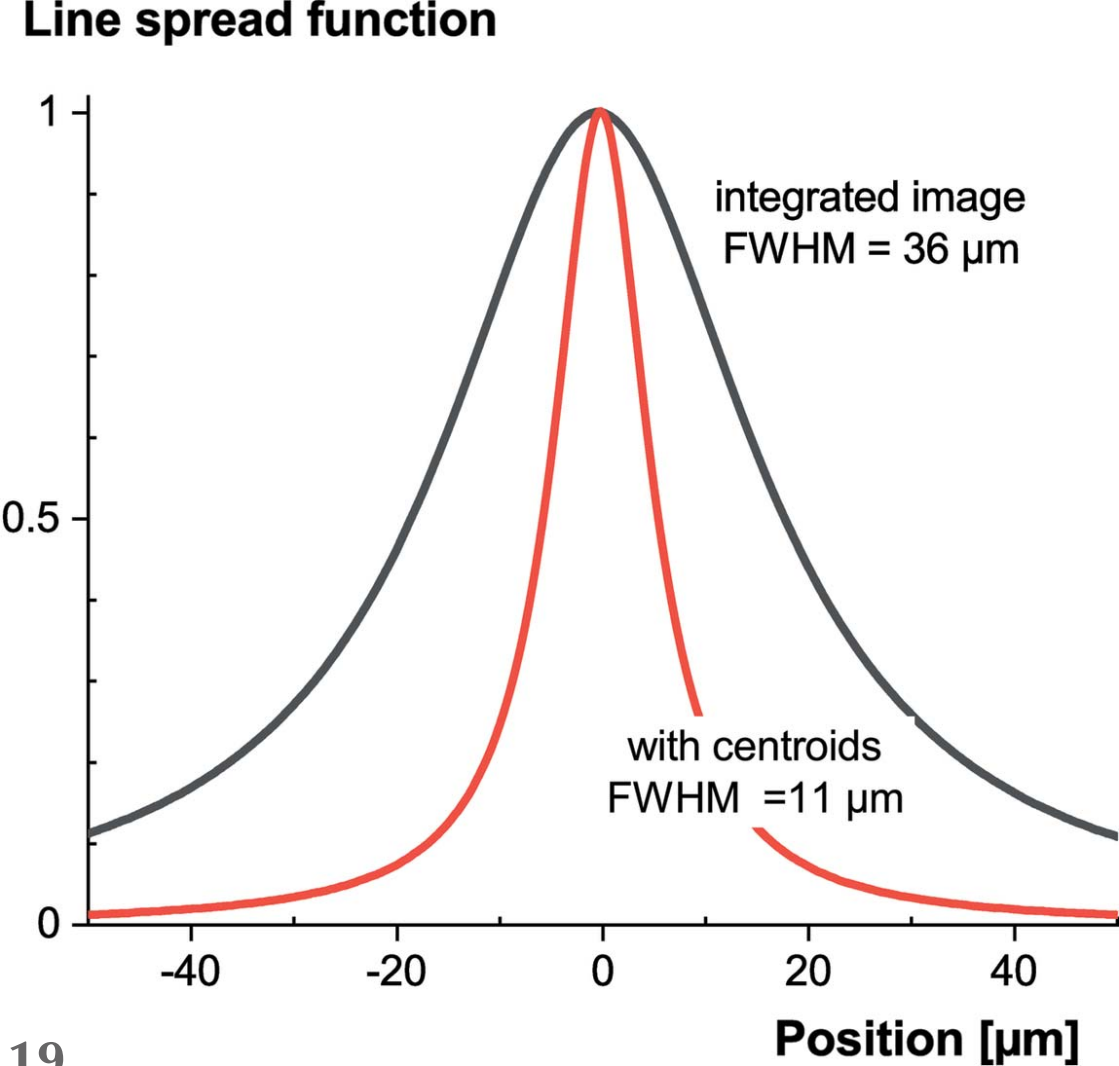
LSF of an 11 µm-thick GOS:Tb scintillating screen with an integrating image and with a reconstructed image based on centroids of single X-ray interactions.

Single X-ray photon identification offers another decisive advantage: it is possible to refine the location of the interaction point. With the light being shared over multiple pixels, the centroid of the light distribution provides a better estimate of the X-ray location than other methods (*e.g.* from the brightest pixel). To confirm this, we measured the LSF of our 11 µm-thick GOS:Tb powder screen with single X-ray photons. Multiple frames (10000) were acquired with a short exposure time and the centroids of the identified single events were used to reconstruct an image. In Fig. 19 ▸, we show that this approach significantly improves the achievable spatial resolution. A similar tripling of the spatial resolution based on the localization of X-ray interactions was observed earlier by O’Connell *et al.* (2020 ▸). Finer charge-cloud localization techniques can further improve it and help in finding a balance between system efficiency and spatial resolution (Gureyev *et al.*, 2014 ▸).

## 6. Conclusions and outlook

Single X-ray photon detection is possible down to 20 keV with the help of widely distributed GOS:Tb powder screens coupled to a commercial camera with a fiber-optics plate. Several parameters can also further be improved. The recent development in the design of sCMOS sensors builds opportunities to assemble cameras with fast frame rates and very low noise levels. The Zyla-HF camera selected for this study offers a low noise level but is limited to a field of view of 16 mm × 14 mm. Larger fields of view are achievable with detectors such as Lassena (Sedgwick *et al.*, 2013 ▸) and CITIUS (Hatsui, 2020 ▸), for instance, but for now they come at the price of higher noise levels. However, new products appearing on the market could soon provide the required performances.

A careful screening of existing scintillators would help in finding the best candidates to assemble an innovative detector combining the advantages of direct and indirect detectors. A line of work that we think offers the best potential added value is to evaluate the performances in single X-ray photon detection of micro-structured scintillating screens (Svenonius *et al.*, 2009 ▸; Sahlholm *et al.*, 2011 ▸; Chen *et al.*, 2018 ▸). The main interest is to better contain the scintillation light. This would allow increasing the thickness of the scintillating layers, thereby increasing the detector efficiency, without the limitations observed with powder screens, which significantly spread the scintillation light over multiple pixels. In parallel, a lot of effort is currently being invested in direct detectors based on high-*Z* materials, *e.g.* based on AsGa and CdTe (Pennicard *et al.*, 2018 ▸; Brombal *et al.*, 2018 ▸; Fiederle *et al.*, 2020 ▸). However, the decisive advantage of indirect detectors is to offer a very uniform and easily scalable field of view at a reasonable cost. Additionally, the versatility allowed by the easy exchange of the scintillating layer makes it possible to virtually tailor the detector to fit any specificity of the applications.

## Figures and Tables

**Table 1:** Camera settings and measured performances

	Single X-ray detection	Light-output measurements
Readout mode	200 MHz – rolling shutter	200 MHz – global shutter
Gain settings	Low noise	High dynamic range
Electronic gain, *K*	3.38 ADU/e	2.17 ADU/e
Readout noise (r.m.s.), σ_d_	1.27 e	2.31 e
Absolute sensitivity, μ_e.min_	1.86 e	2.87 e
Saturation capacity, μ_e.sat_	1005 e	26400 e

**Table 2:** Characteristic emission lines and *K*-edge energies from Arndt *et al.* (2006 ▸) with the selected fluorescent targets and incident-beam energy

	*K*-series energies (keV)						
Target material	*K*α_2_	*K*α_1_	*K*β_3_	*K*β_1_	*K*β_2_	*K*-edge energy (keV)	Beam energy (keV)
Cu	8.028	8.048	8.903	8.905	–	8.993	9.5
Ag	21.991	22.163	24.912	24.943	25.463	25.531	26.0
Sb	26.110	26.359	29.677	29.725	30.402	30.499	31.0
Gd	42.309	42.996	48.554	48.696	49.964	50.207	51.0
W	57.982	59.318	66.951	67.244	69.100	69.517	70.0

**Table 3:** Thickness, density and X-ray absorption of the GOS:Tb scintillating screens (the X-ray generator settings are detailed in Section 2.2)

Thickness (µm)	Density (g cm^−3^)	X-ray absorption at 15.4 keV (%)
11	3.82	24
21	4.17	43
27	4.55	51
29	4.99	58
47	4.87	73

**Table 4:** Light output of the GOS:Tb scintillating screens measured at 15.4 keV (the X-ray generator settings are detailed in Section 2.2)

Thickness (µm)	Signal per X-ray	Average energy deposit (keV)	Light output (ph keV^−1^)
11	54 ADU, 126 ph	3.60	34.3
21	96 ADU, 223 ph	6.32	33.6
27	110 ADU, 256 ph	7.95	32.4
29	130 ADU, 303 ph	8.62	33.4
47	148 ADU, 345 ph	11.0	30.3

**Table 5:** Single X-ray photon sensitivity thresholds (with four pixels above the noise) with GOS:Tb scintillating screens and a camera pixel size of 6.5 µm

Screen thickness (µm)	Light output (ph keV^−1^)	Light fraction, η_min_ (%)	Collected signal (e keV^−1^)	Absolute sensitivity (e)	Single-photon threshold (keV)
11	36.3	0.76	0.061	1.86	30.6
21	35.4	0.77	0.060	1.86	31.0
27	32.0	0.69	0.049	1.86	38.2
29	34.1	0.60	0.045	1.86	41.3
47	30.2	0.41	0.027	1.86	68.2

**Table 6:** Single X-ray photon sensitivity thresholds (with four pixels above the noise) with GOS:Tb scintillating screens and a camera pixel size of 26 µm

Screen thickness (µm)	Light output (ph keV^−1^)	Light fraction, η_min_ (%)	Collected signal (e keV^−1^)	Absolute sensitivity (e)	Single-photon threshold (keV)
11	36.3	4.61	0.369	7.44	20.2
21	35.4	5.08	0.396	7.44	18.8
27	32.0	4.91	0.346	7.44	21.5
29	34.1	4.65	0.349	7.44	21.3
47	30.2	3.79	0.252	7.44	29.5
